# SDN-Oriented 6G Industrial IoT Architecture Design and Application to Optimal RIS Placement and Selection

**DOI:** 10.3390/s26020411

**Published:** 2026-01-08

**Authors:** Francesco Chiti, Matteo Lotti, Sara Picchioni, Laura Pierucci

**Affiliations:** Department of Information Engineering, University of Florence, 50139 Florence, Italy

**Keywords:** 6G architecture, Industrial Mobile IoT, OpenFlow-based SDN, RIS modeling, RIS placement and selection

## Abstract

This paper presents a high-level system architecture that integrates the Software Defined Networking (SDN) paradigm in 5G/6G networks with the aim of supporting the requirements expected for Industrial Internet of Things (IIoT) devices and services. To this purpose, we include multiple Reconfigurable Intelligent Surfaces (RISs) systems and provide for them an abstract representation consistent with the OpenFlow interface and messaging framework. The main contribution of this is firstly focused on designing a comprehensive framework that specifies the modules, components, interfaces, protocols, and message exchanges across the typical three layers SDN architecture. In addition, we characterize the Network Discovery (ND) and Host Discovery (HD) protocols that enable the SDN Controller to achieve a global and updated view of the network. Then, the RIS Placement and Selection Problem (RPSP) is formulated by using two graph-theory approaches, i.e., Set Covering (SC) and Minimum Spanning Tree (MST). Finally, we conduct an extensive simulation campaign that evaluates the performance of the discovery phases and the RIS placement/selection algorithms in realistic industrial environments. The results highlight the advantages achieved in terms of coverage and complexity.

## 1. Introduction

### 1.1. Context and Motivation

The rapid advancement in mobile communications is driving research towards Sixth Generation (6G) technology, a paradigm shift aimed to surpass the performance limits of 5G and unlock radically novel use cases [1,2]. 6G aims to deliver peak data rates in the Tbps range, ultra-low latencies (μs), and enable Smart Connectivity that seamlessly integrates the physical and digital worlds [3]. This future is strongly tied to the development of next-generation industrial and mobile systems, often termed Internet of Things 2.0 (IoT2.0) [4], where machine-to-machine (M2M) seamless communications and automatic management between heterogeneous devices can be enabled without human intervention. This has the potential to revolutionize industrial processes and provide significant benefits to society through fully intelligent and automated remote management systems.

Achieving the ambitious goals set for this evolution necessitates a fundamental redesign of the network architecture and the introduction of groundbreaking technologies [5]. Mobile 5G communication technologies have served as pivotal enablers for the deployment and operational scaling of IoT networks and applications. The 5G standard provided substantial enhancements to the IoT ecosystem through its inherent service features, including Enhanced Mobile Broadband (eMBB), Massive Machine-Type Communication (mMTC), and Ultra-Reliable and Low-Latency Communication (URLLC), resulting in optimized service provisioning characterized by high throughput, low latency, and energy efficiency [6]. However, the unprecedented growth in smart devices and the escalating demand for advanced technical criteria (e.g., autonomous, ultra-large-scale, highly dynamic, and fully intelligent services) are now demonstrating the limitations of the current 5G capabilities. The rapid expansion of complex, automated IoT systems, such as remote robotic operations, is projected to exceed the capacity of existing 5G wireless frameworks [7,8].

Consequently, research attention has shifted toward 6G wireless networks, which will address 5G’s technical shortcomings. 6G is, indeed, envisioned to deliver a paradigm shift in IoT service quality via superior features: ultra-low-latency communications, extremely high throughput, satellite-based service delivery, and massive autonomous network architectures [9]. In particular, these requirements will accelerate the 6G-based IoT deployments across all operational domains, including data sensing, device connectivity, wireless communication, and advanced network management [10].

To this purpose, the deployment of Reconfigurable Intelligent Surfaces (RISs) represents a highly promising technological advancement for 6G networks, specifically addressing inherent propagation constraints and improving coverage. An RIS consists of a near-passive device composed of a massive number of elements capable of signal manipulation, thereby reflecting and steering the incident electromagnetic waves. This capability fundamentally shifts the wireless medium from a limiting factor to an assisting entity in communication systems [11]. RIS integration is explicitly identified as a crucial enabling technology for next-generation 6G IoT, providing indispensable support for coverage extension in systems operating at millimeter-Wave (mmWave) and Terahertz (THz) frequencies, and enabling dynamic network optimization in scenarios, such as fixed or quasi-static industrial environments [12]. Due to their reconfigurability, RISs are viewed as a compelling approach for wireless system design and optimization, as they facilitate favorable signal propagation, and acquisition, thus creating a smart radio environment highly beneficial for 6G applications. In multi-cell IoT networks, for instance, RISs can simultaneously bolster signals from serving base stations, while mitigating inter-cell interference across a massive number of devices. Furthermore, RISs offer significant potential for enhancing data offloading rates to edge servers. Since offloading data volume is heavily dependent on channel gain, RISs can establish virtual array gain and reflection-based beamforming gain on offloading links, allowing for more time-efficient data processing at the edge compared to traditional methods. However, the effective use of multiple RISs requires a coordinated and scalable control mechanism capable of managing their real-time configuration, thereby highlighting the need for an advanced architectural paradigm that can provide global optimization and dynamic programmability. Since the traditional network architectures are deemed insufficient, the adoption of Software Defined Networking (SDN) emerges as an indispensable enabling solution [13].

SDN is an innovative approach to design, implement, and manage networks by decoupling the data forwarding from the control itself. This separation makes network administration significantly more flexible, since both planes can operate independently: the Data Plane (DP) is solely responsible for packet forwarding, while the Control Plane (CP) provides the “intelligence” for optimizing routing paths, setting priorities, and defining routing policy parameters. The SDN Controller monitors the network and summarizes its features using a global view to implement services and policies, all while delegating traffic forwarding to DP network devices. This paradigm allows centralized programmability and abstraction of the underlying infrastructure for applications and services. This flexibility is crucial for coordinating resource allocation, addressing the latency challenges of the 5G/6G CP, and is essential for dynamically orchestrating and optimizing the configuration of RIS elements based on real-time Industrial IoT (IIoT) needs [13].

As previously addressed, the SDN architecture usually consists of the DP, CP, and Application Plane (AP), where Controller is the fundamental component. Indeed, it maintains a global view of the network and, according to the service requirements, it handles the needed traffic flows by configuring forwarding devices with proper control message exchanging.

The SDN Controller typically has a modular architecture, which enhances its flexibility and scalability. The core modules often include *Topology Manager*, responsible for discovering and maintaining an up-to-date map of the network; *Notification Manager*, which receives, processes, and forwards notifications to an application; *Device Manager*, responsible for the configuration of SDN Switches and the management of the flow table, and *Forwarding Path Optimizer*, which uses routing information collected from switches to determine paths. DP is instead comprised of SDN Switches responsible for sending data in accordance with the forwarding rules set by the SDN Controller.

Within this broader vision, this paper focuses on an IIoT context requiring a network infrastructure to support a massive number of heterogeneous devices, real-time applications, and high-precision autonomous operations [14]. In particular, advanced automation, industrial digital twins, and high-accuracy remote control require in the IIoT domain extremely stringent reliability and latency [13]. This is a formidable challenge in highly dynamic RIS-enabled environments, demanding an intelligent control [15].

### 1.2. Background and Open Challenges

Recent scholarly work has started to embed RIS technology into various 6G-based IoT use cases. Research demonstrates that RISs can be applied in scenarios such as smart buildings [16], where they facilitate the interface between indoor and outdoor entities to ensure seamless household access, offering enhanced spectral efficiency and cooperative interference avoidance. Additionally, RISs have been incorporated into Vehicular IoT (VIoT) networks, specifically in vehicle-to-vehicle (V2V) communications, utilizing RIS-based access points and relays for coordination [17]. The application of RIS also extends to radio-frequency (RF) sensing, supporting tasks like human posture recognition in surveillance and remote health monitoring [18]. By periodically programming the RIS configurations, optimal propagation links are established, generating multiple independent paths that accumulate valuable posture information, leading to significantly enhanced recognition performance compared to random or non-configured setups. Despite significant technological advances, the proper integration of RIS within an SDN paradigm presents several open challenges that the current state-of-the-art has yet to fully address.

Although envisioned in the seminal paper [19], the concept of programmable wireless environments (PWEs) was firstly introduced by Liaskos [20] to map a wireless component into a software-controlled resource. Even though the paper focuses on only the metasurface hardware and software, a preliminary SDN foundations is addressed to abstract the physics behind PWEs to manipulate them in an algorithmic way and to achieve scalable inter-networking with multihop communications and a logically centralized control.

Further, the RIS adoption for dynamically setting up a 6G wireless backhaul system managed by a SDN Controller is addressed in [21]. However, the article only investigates the factors that influence RIS realization and application, such as gain, far-field, losses, and communication metrics.

The design of a programmable software-defined optical wireless communication-based Autonomous Aerial Vehicles (AAVs) networking platform is addressed in [22], together with preliminary performance evaluation results and the possible extension toward the full adoption of RIS.

However, a significant gap remains in the literature as a viable integration of software-controllable RIS within flexible, programmable network architectures is not yet fully addressed, while it represent a crucial requirement for modern IIoT. The only partial contribution towards this goal is represented by [23], where the authors propose re-designing the 6G control plane by moving signalling handling out of the core to improve modularity and flexibility for controlling complex 6G elements like RIS.

Nevertheless, even though SDN provides in principle the necessary network programmability, integrating the control loop for physical-layer elements like RIS in real-time is non-trivial. The open challenge lies in designing a resilient and fast control plane that can handle the massive signaling overhead associated with RIS configuration (the physical layer) while simultaneously meeting the ultra-low latency requirements imposed by time-sensitive applications (the application layer).

### 1.3. Proposal and Main Contributions

Differently from the discussed literature, and refining the approach proposed in [15], this article proposes a high-level, system-wide architecture, where the SDN paradigm is integrated within the 6G cellular architecture and specialized for IIoT devices and services. Indeed, to the best of our knowledge, no existing work has simultaneously addressed the challenges of (i) introducing SDN-based control architecture and protocols and (ii) integrating RIS devices within this framework. Our proposed architecture and optimization framework are the first to jointly consider these elements, closing a critical gap in the research landscape.

To address this problem, the paper initially outlines a comprehensive system architecture, detailing the constituent modules, components, interfaces, protocols, and messages exchanged within the Host, Data, Control, and Application Planes, according to the usual SDN architectural paradigm.

As a consequence, the focus is on DP devices architecture, which in our case are mainly represented by RISs. Accordingly, they have been characterized using an abstract model and rendered congruent with the OpenFlow interface and messaging framework.

This framework is then applied to system management, specifically describing the phases of Network Discovery (ND) and Host Discovery (HD), along with designing the related protocol.

Following this, the article further characterizes the problem of RIS Placement and Selection. To this end, two distinct approaches rooted in graph theory are proposed, i.e., the Set Covering (SC) and Minimum Spanning Tree (MST) algorithms, since they align with the network view provided by the SDN Controller after the discovery phases.

Finally, an extensive simulation campaign investigates the performance of both the discovery phases and the placement and selection process itself, demonstrating the effectiveness of the proposed overall system in real-world industrial scenarios.

The remainder of this paper is organized as follows. Section 2 presents the proposed SDN-oriented system architecture in terms of overall architecture, modules, and interfaces. In addition, the RIS abstracted operational model is properly characterized, together with a specific protocol designed to discovery networked devices along Data or Host Planes. The RIS Placement and Selection Problem is then pointed out following an integrated formulation, and a couple of graph-based strategies are proposed for addressing an effective and joint solution. Finally, Section 3 investigates the related performance by first setting up a specific software framework and then providing numerical results and discussion, while Section 4 concludes the work and suggests future research directions.

## 2. Proposed System Design

### 2.1. SDN-Oriented Architecture, Modules, and Interfaces

The integration of the SDN paradigm and RIS technology is a cornerstone of 6G innovative network architecture, enabling unprecedented levels of efficiency, flexibility, and adaptability. Together, SDN provides real-time network programmability and optimization, and RIS acts as a cost-effective, energy-efficient mean to extend coverage and improve spectral efficiency. This synergy is pivotal for creating smarter, adaptive, and context-aware networks that meet the challenges of next-generation IoT systems.

In addressing the integration and control of RIS through SDN Controller we rely on the OpenFlow interface and protocol. OpenFlow is a standardized and open solution facilitating communication between the SDN Controller and Switches. It operates exploiting flow tables that define how packets are processed and forwarded, alongside APIs that exchange messages between SDN Switches and Controllers. OpenFlow employs Transport Layer Security (TLS) protocols for secure communication and uses various table types (flow, group, and meter tables) to manage traffic effectively. According to our implementation, each packet traverses the flow tables pipeline, executing specific actions such as output, queue assignment, or modifications, as determined by the matching rules.

**Figure 1:**
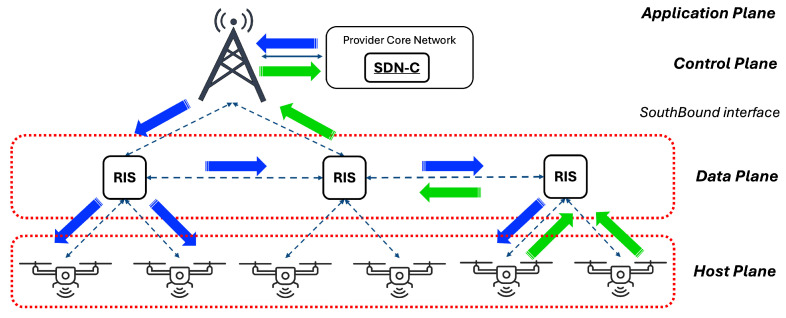
Proposed Multi-layer SDN-oriented IIoT architecture for RIS integration, with the indication of upstream (green) and downstream (blue) flows.

Figure 1 shows the multi-layer structure of SDN-RIS integration. At the lower level, the Host Plane (HP) includes the IIoT terminals, which act as both data sources and destinations. The figure also illustrates the possible downlink (blue) and uplink (green) flows between the devices in the architecture.

The upper level, i.e., DP, incorporates RIS. To seamlessly integrate RISs into the SDN framework, they are abstracted as OpenFlow Switches. This is accomplished by wrapping them into an abstract data structure: the process and the associated benefits are detailed in the following. In addition, the SDN Controller is included in the CP that is interfaced with DP via the OpenFlow protocol. According to our view, the proposed Controller is a virtual process integrated in a physical server, typically in the Provider Core Network (CN) to be compatible with the already-existing provider SDN framework (without loss of generality, the SDN Controller could be installed also in a Mobile Edge Computing (MEC) server directly connected to the gNodeB via a wired link, especially for low latency scenarios), and logically connected with the deployed RIS (this additionally requires that RISs are uniquely IP addressed and are capable of packet header fields parsing). As previously introduced, the SDN Controller comprises various modules, such as the Device Tracker and Topology Manager, with planned enhancements to include a Path Manager and Optimizer to define and manage traffic forwarding rules. At the topmost level, the AP includes applications and services with specific requirements. Accordingly, the SDN Controller configures the underlying network to meet these application-driven needs. Our case study typically focuses on IIoT industrial services.

The approach adopted in this specific domain relies on accomplishing a generic RIS within an OpenFlow compatible Switch model in order to seamlessly integrate the RIS into a SDN framework. This design choice enables centralized, programmatic control over the RIS device using standard SDN control protocols, specifically OpenFlow, via the SDN Controller. By wrapping the RIS within an OpenFlow Switch we effectively expose its control interface to the SDN CP, simplifying orchestration and interoperability.

### 2.2. RIS Operational and Abstracted Models

In order to integrate an RIS into the proposed architectural framework, it is essential firstly to characterize RIS operational physical principles and, accordingly, to derive a general model. Fundamentally, an RIS operates by receiving an incoming electromagnetic signal, applying specific signal processing, such as phase shifts or amplitude adjustments, and then retransmitting the modified signal. This process is achieved through a set of predefined receive and transmit beams, each characterized by a specific angle relative to the RIS surface, which determines the directionality of signal reception and reflection. For a coherent and gradual development of the RIS, several possible models with increasing complexity have been considered and integrated to investigate a trade-off between performance and feasibility, as in the following:

1. **Amplifier**: “The first model is the basic RIS version in which it is only required to reflect the received signal through a single output port. This enables signal redirection without phase control, but only power gain. Once the signal is received, it can be retransmitted exclusively through a predetermined fixed beam direction. Moreover, since this version has no embedded control logic, it lacks the capability to generate or initiate signals autonomously; it can only passively retransmit the signals it receives. Consequently, the network discovery phase must be initiated by gNodeB, which transmits a signal that is then reflected by the RIS elements. From a performance perspective, this model offers clear advantages in terms of simplicity, cost, and power efficiency. Its completely passive nature allows extremely low-power operation, making it attractive for large-scale deployments, where energy constraints are critical. However, these benefits come at the cost of severely limited functionality: since it is unable to modify the reflection direction, to support multi-user scenarios, or to adapt to mobility, it significantly restricts the applicability of this RIS type in dynamic wireless environments.”
2. **Amplifier with Phase Shifter**: “In this second case, RIS is equipped with a microcontroller for beamforming control. It supports single-beam operation with a discrete phase set and functions as a multi-port hub. Once the signal is received, it may be retransmitted over a single beam and selected from a discrete set of predefined beam directions; however, only one beam can be active at any given time. This architectural model provides a remarkably wider operational set. Indeed, the ability to select a beam direction in real time improves coverage and enhances its suitability for dynamic environments. At the same time, the design remains relatively lightweight, maintaining a reasonable balance between performance, cost, and power consumption. Nevertheless, the single-beam constraint limits its effectiveness in multi-user scenarios. In this case, the network discovery protocol can be initiated by the RIS itself.”
3. **Multi-beam Amplifier with Phase Shifter**: “This third model, which brings RIS to maturity, further extends capabilities by supporting multi-beam configurations, managed via OF Group Table mechanisms. Once the signal is received, it can be simultaneously retransmitted across multiple beams, enabling multi-directional signal propagation. This advanced model offers substantial benefits in terms of flexibility and performance. The ability to serve multiple users simultaneously and to shape the propagation environment in complex ways significantly enhances network capacity and coverage. However, these capabilities come with increased architectural complexity, higher cost, and greater power consumption, as well as a heavier reliance on synchronization and control-plane signaling between the RIS and the SDN Controller.”

**Figure 2:**
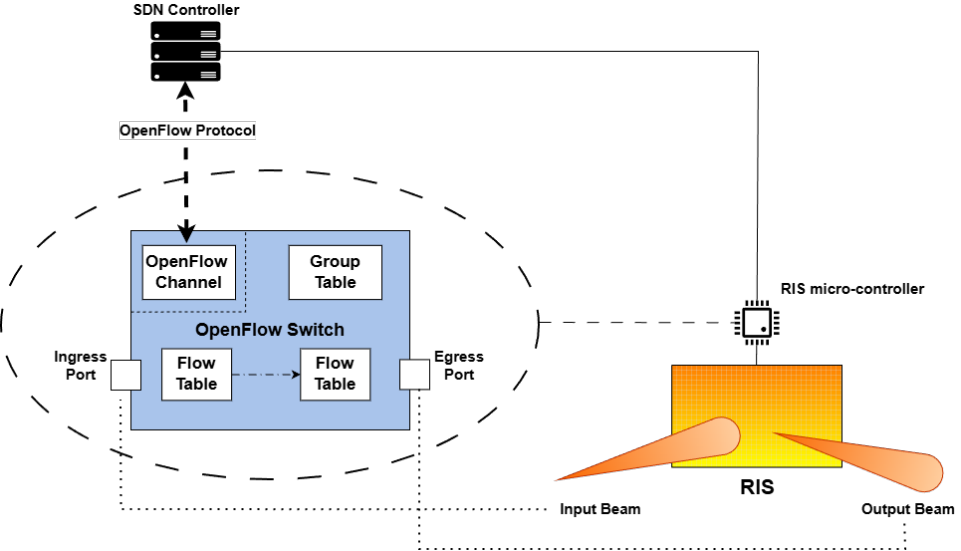
Mapping of RIS into an OpenFlow Switch.

It is worth pointing out that, for the integration of the RIS into an SDN control framework, it is required that RIS is equipped with an onboard micro-controller able to install OpenFlow library and perform as an SDN Switch for match-and-action operation. In addition, some mappings are necessary, as an OpenFlow Switch port needs to be matched with the RIS potential beams. In the proposed architectural paradigm, each RIS is therefore abstracted as an SDN Switch, where beams correspond to input/output ports, as shown in Figure 2. This abstraction allows the SDN Controller to configure and manage RIS behaviors through standard OpenFlow operations. By mapping beams to SDN Switch ports, the Controller can dynamically configure RIS beam patterns by simply programming the appropriate ports and enabling fine-grained and flexible control of the RIS functionality through standard SDN operations, as usually expected [13]. This setup facilitates the assessment of RIS interoperability and its impact on network behavior in complex industrial scenarios. According to the proposed abstraction, each physical RIS operating in re-radiation modes to transmit or receive signals corresponds to *virtual* Switch, whose *virtual* forwarding port represents each RIS beam, such that a direct correlation exists between the physical beams and the *virtual* ports. The SDN Controller interacts with these Switches and is aware only of the active ports on each Switch and not of the overall possible beams of the RIS. This abstraction ensures that the Controller does not handle any signal manipulation, while, instead, it simply defines forwarding rules.

### 2.3. Network Discovery Protocol

*Network Discovery* (ND) is a critical process in an SDN system since it provides the Controller with a comprehensive view of the network topology. This process involves identifying network devices, their interconnections, and other key attributes. ND protocol is an essential component of this mechanism, allowing the Controller to dynamically update its abstract representation of the underlying system as devices join, leave, or change state (it is worth pointing out that in cases of static RIS deployment and configuration the ND could not be necessary). The discovery process usually performed in a OpenFlow oriented system relies on protocols such as Link Layer Discovery Protocol (LLDP). LLDP is a standard approach (IEEE 802.1AB [24]) in which devices broadcast information to directly connected neighbors. However, in an SDN environment, the SDN Controller forces SDN Switches to send out LLDP packets over their ports. These packets contain information such as the SDN Switch identifier, port number, and other metadata. Upon receiving a packet, the SDN Controller configures a Switch to forward the information back to it so that it is possible to derive the network topology. In particular, a global abstract network view, capturing the nodes and their interconnections via an expanded (hyper)graph, is set-up and maintained.

As detailed in Section 3.3.1, the overall network discovery mechanism can be conceptually divided into two complementary phases: Switch Discovery (SD) and Host Discovery (HD). SD focuses on identifying the inter-switch links by analyzing LLDP-based signaling. In our reference scenario, each time a link between two RIS nodes is detected, the Controller adds a corresponding edge to the topology graph, associating it with a weight related either to (i) the Received Signal Strength Indicator (RSSI), or (ii) the packet delivery delay. HD, instead, concerns the identification and tracking of end devices connected to the network, and it is usually more simple as it relies on typical cellular procedures. This process is typically performed proactively by the SDN Controller, for example, by leveraging periodic packets sent by the UEs or by triggering specific probing mechanisms. The information obtained in this phase complements SD, allowing the Controller to maintain a complete and consistent representation of both the infrastructure nodes and the end devices.

### 2.4. RIS Placement and Selection Problem

Optimizing the RIS positioning is crucial for reducing installation costs, since their proper placement allows us to achieve better network performance with minimum complexity, especially if compared to a dense or even random deployment. By taking into account the propagation scenario, RISs can be placed in particular locations, where they can maximize signal enhancement or redirection. In essence, an optimized RIS deployment ensures cost-effectiveness by balancing performance and infrastructure set-up and maintenance costs. In addition to that, once a set of RISs has been installed, they can be activated on demand and configured to handle data flows according to the specific QoS requirements.

This motivates the RIS Placement and Selection Problem (RPSP) formulation, where RPSP has its focus on both optimal geometrical placement of RIS antennas within a specific area, together with RIS dynamic selection and activation, potentially aiming at improved data flows management. In order to provide a unified RPSP statement, we introduce the following variables:

- $U≐\{u_{i}\},\;i=1,\ldots ,I$: the set of positions of UEs to be connected;
- $R≐\{r_{j}\},\;j=1,\ldots ,J$: the set of locations for RIS and gNB deployment;
- $C≐\{c_{ij}\}$: the matrix of cost to connect the *i*-th UE with the *j*-th RIS (or gNodeB);
- $X≐\{x_{ij}\}$: the decision matrix, where the element $x_{ij}=1$ if the *i*-th UE is assigned to the *j*-th RIS (or gNodeB), and 0 otherwise.

The joint optimization problem can be expressed as:

(1) $$\begin{array}{cc} \min\; & \sum_{i=1}^{I}\sum_{j=1}^{J}c_{ij}x_{ij} \end{array}$$

(2) $$\begin{array}{cc} s.t.\; & ∥X∥\geq pI\;,\;p\in [0,1] \end{array}$$

(3) $$\begin{array}{cc} \; & R\;\mathrm{is}\;\mathrm{connected} \end{array}$$

where the objective function to be minimized in (1) is the total assignment cost subject to typical connection constraints, i.e., (2) unique (p=1) or incomplete assignment (p<1), and (3) every couple of elements ∈R are internally connected.

It can be noticed that in the RIS placement U and R are expressed in terms of probability distributions, and it is not possible to asses the connection cost so that $c_{ij}=1,\;\forall i,j$. Conversely, for the RIS selection U and R are exactly known, together with the connection cost C, p=1 and (3) is automatically satisfied by the solution of the RIS placement.

In order to address the solution of our optimization problem, we resort to a graph- theoretic framework, since it has been proven to be effectively applicable to network deployment and planning [25], highlighting the potential of using graph-based models to optimize coverage and connectivity in complex network scenarios. Moreover, in [26], a connectivity-aware approximation algorithm for relay node placement in wireless sensor networks is proposed, combining a local Set Covering (SC) approach to ensure full coverage of sensor nodes with a Minimum Spanning Tree (MST) heuristic to maintain network connectivity, hence demonstrating how the integration of coverage and connectivity optimization can effectively guide the deployment of network devices.

Motivated by prior works, we adopt the following strategies in our study for addressing RPSP:

- *Set Covering (SC)* was explored in order to select an optimal subset of active RIS elements so that all UEs in the environment are effectively covered by at least one beam. The SC problem, in general, involves selecting the smallest number of sets from a collection such that their union includes every element in a target set. In this context, each RIS is associated with a set of UEs it can cover, and the algorithm selects the smallest subset of RISs, whose combined coverage serves all the users. This approach helps to reduce the number of RISs required, leading to a lower deployment cost, while maintaining comprehensive signal coverage.
- *Minimum Spanning Tree (MST)* has been employed to further optimize the RIS-UE coverage, and, thus, is more related to RIS selection. In this case, the network is modeled as a weighted graph, where edges represent possible coverage links between RIS elements and UEs, and weights correspond, for instance, to the involved communications delay. The MST is then used to select a subset of these links that connect all UEs to RIS nodes with minimal overall cost, ensuring that every UE is reachable through the most efficient path traversing a set of RISs.

The first approach we proposed relies on a simple preliminary heuristic, which iterates over the set of considered RISs (see Algorithm 1). For each RIS, it identifies the users that can be reached; if a user has not yet been assigned to any RIS, it is associated with the current one. Consequently, the first RIS serves all users within its coverage area, while the second RIS serves only those reachable users that have not already been assigned to the first RIS. Further, this algorithm is iterated over all the remaining RISs. This approach could be assumed as a worst-case benchmark, allowing a comparison with the more optimized strategies, based on the SC formulation and on the MST approach.

The SC approach is grounded in Set Theory and, in order to be applied to our scenario, it requires defining the set of all possible RISs within the environment and, for each RIS, the subset of users it can reach and thus serve. This is achieved by exploiting the spatial positions of both the RIS and the IoT devices: for each RIS, it is verified whether the IoT node positions fall within one of its feasible beams. As a result, we obtain the set of IoT devices served by each RIS. By applying the SC algorithm in this way, we can then determine the minimum number of RIS needed to cover the maximum number of IoT devices. The SC approach is then used to select the RISs from an already-defined set and place them within the scenario (see Algorithm 2).
**Algorithm 1:** Heuristic-based RIS Selection Procedure
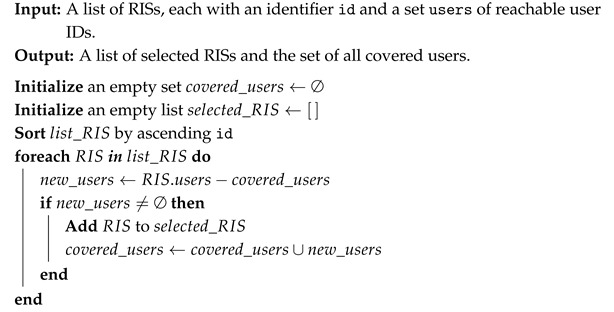


**Algorithm 2:** Set Covering-based RIS Selection Procedure

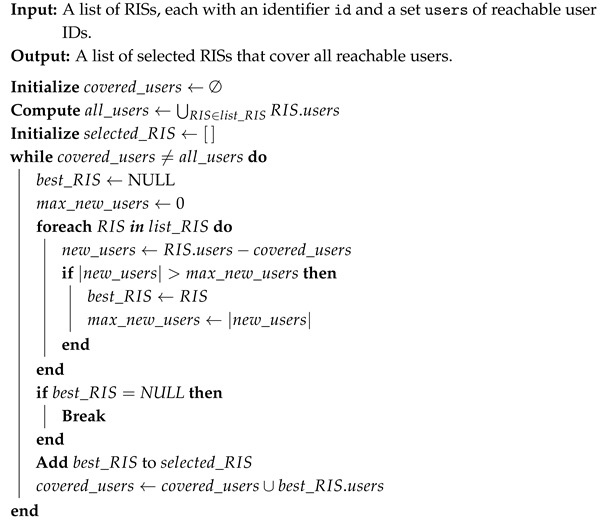



**Figure 3:**
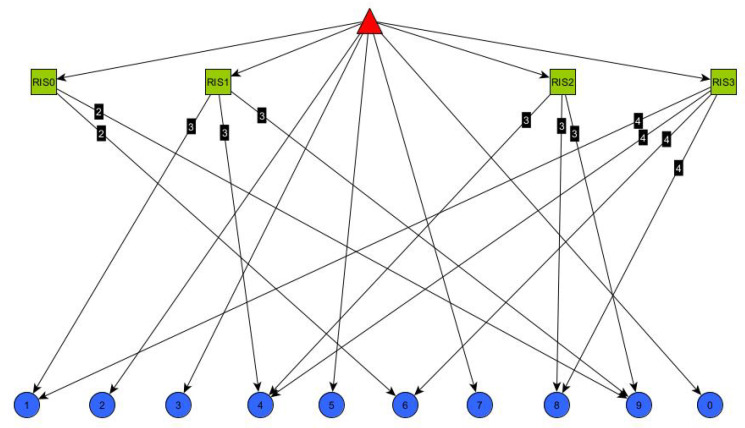
Example of network topology with RIS fan-out values annotated on the edges.

Finally, the MST approach, on the other hand, pursues a different goal: identifying the minimum-cost path among the nodes already arranged in a tree fashion. In our case study, a graph representing the network topology is constructed, including the gNodeB, RISs, IoT devices, and all their links. Moreover, the edges connecting RISs and IoT devices are labeled with a weight. In our approach, we adopted the fan-out of each RIS as a proper cost metric, since it is proportional with the RIS load (i.e., how many connections a RIS maintains with the served users) and representative for the processing and data delivery delay, especially for RIS simple models, where it can only serve one user at a time (see Figure 3).

Our MST-based algorithm ensures that for each IoT device, the minimum weight edge connecting it to a specific RIS is selected, while the remaining edges are removed. The fan-out of the unselected RISs is then recalculated accordingly (see Algorithm 3).
**Algorithm 3:** MST-Based RIS Selection with Dynamic Weight Update
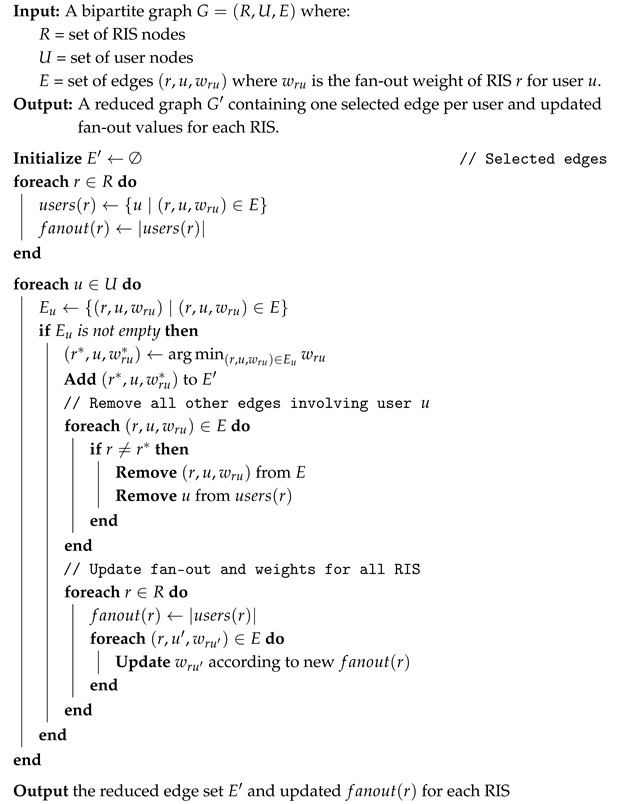


It is worth noticing that the last two algorithms (Algorithms 2 and 3) could be intended as complementary: SC provides, indeed, efficient spatial coverage, while MST ensures efficient connectivity, both contributing to the optimal and cost-effective resolution of RPSP. In our proposal, we assumed that the RISs were placed at predefined locations; therefore, selecting a specific RIS inherently determines its position (an alternative approach could first identify the possible location for RIS placement, within which a particular RIS can then be properly deployed and configured exactly when and where it is needed to support communication). Furthermore, the SC algorithm can be regarded as a preliminary procedure executed before the actual communication phase, aimed at selecting, placing, and configuring the required RISs. Conversely, the MST algorithm can be successively applied to optimize the existing network infrastructure and perform a more efficient selection of RISs to support ongoing data flows.

## 3. Performance Evaluation

### 3.1. Simulation Framework Design

In order to simulate the integration of RIS with the SDN paradigm within the reference scenario, several core classes have been implemented and integrated within an object-oriented, event-based Python 3.12 framework, designed to simulate message exchanges between a gNodeB and UEs.

In particular, the specific classes and features are the following:

- Controller class is responsible for network intelligence and decision-making. It maintains the network topology and a list of connected Switch instances. Communication with the network is performed via instances of the OpenFlowChannel class, which handles the sending and receiving of control messages. Therefore, the OpenFlowChannel represents the Southbound interface between the SDN controller and RISs.
- Switch represents a programmable network node that keeps track of connected UEs (ue_reached) and nearby RIS elements (ris_reached).
- Switches can generate packets (create_packet_in), respond to feature requests from SDN Controller (create_feature_reply), and finally forward data via specific ports (send_on_ofdm_channel).
- RIS class models the RISs, storing their coordinates and beam configurations, and includes a method to determine if a device is within a beam. As mentioned before, communication between SDN Switches and the SDN Controller is handled through instances of the OpenFlowChannel class, which provides methods for sending and receiving control messages.
- UE class represents a user device and contains its position and PUCCH transmission status, with the ability to generate uplink messages (generate_pucch).

The Controller also exposes a Northbound interface to support higher-level modules, such as host tracking and security, allowing external applications to interact with the exposed services. These applications are responsible for defining high-level network behavior, policies, and service logic, while the Controller translates those requirements into low-level configurations that are enforced across the underlying network infrastructure.

### 3.2. Scenarios Characterization

**Figure 4:**
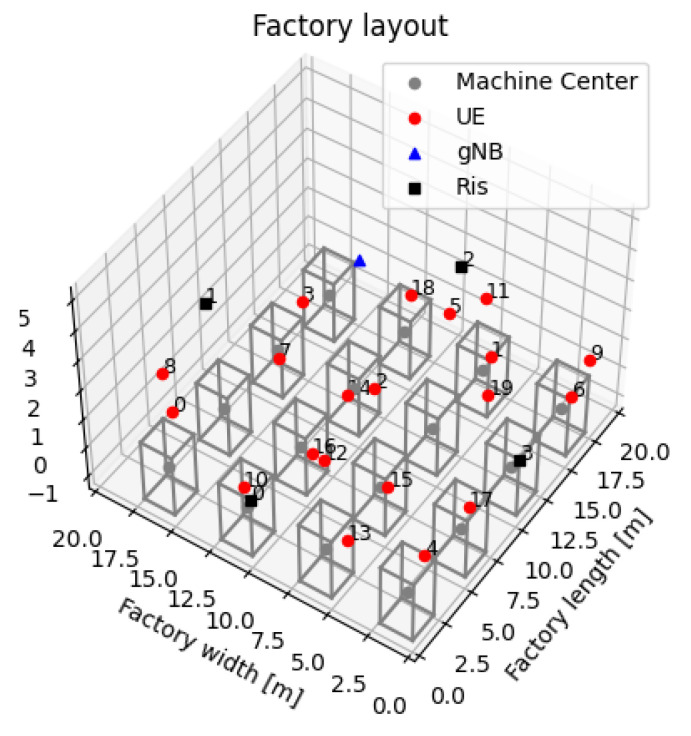
Example of a simulated industrial scenario.

The simulated industrial scenario represents a factory environment structured as a grid of machine units. Each machine is represented by a cuboid, with its center marked by a grey dot. Within this environment, multiple UEs, shown as red circles, are uniformly placed. A single gNodeB, represented by a blue triangle, acts as the central base station coordinating the network. Additionally, several RISs, indicated by black squares, are strategically positioned around the factory layout, as sketched in Figure 4. Furthermore, Table 1 shows the main simulation setting parameters.

The industrial environment has been selected as the reference scenario because it represents one of the most challenging and representative use cases for 6G-enabled Mobile IoT. Industrial facilities typically exhibit harsh propagation conditions, including large metallic surfaces, complex layouts, and frequent NLoS situations caused by machinery, storage racks, and moving equipment. These characteristics make them a natural candidate for evaluating the effectiveness of RIS-assisted communications. Furthermore, the need for high reliability, extended coverage, and flexible reconfigurability aligns well with the advantages provided by SDN-controlled RISs.

### 3.3. Numerical Results

In the following, we present the tests and simulations performed to evaluate the proposed approach. The experimental setup, methodologies, and parameters used are described in detail, followed by a comprehensive discussion of the obtained results.

#### 3.3.1. Network Discovery

**Figure 5:**
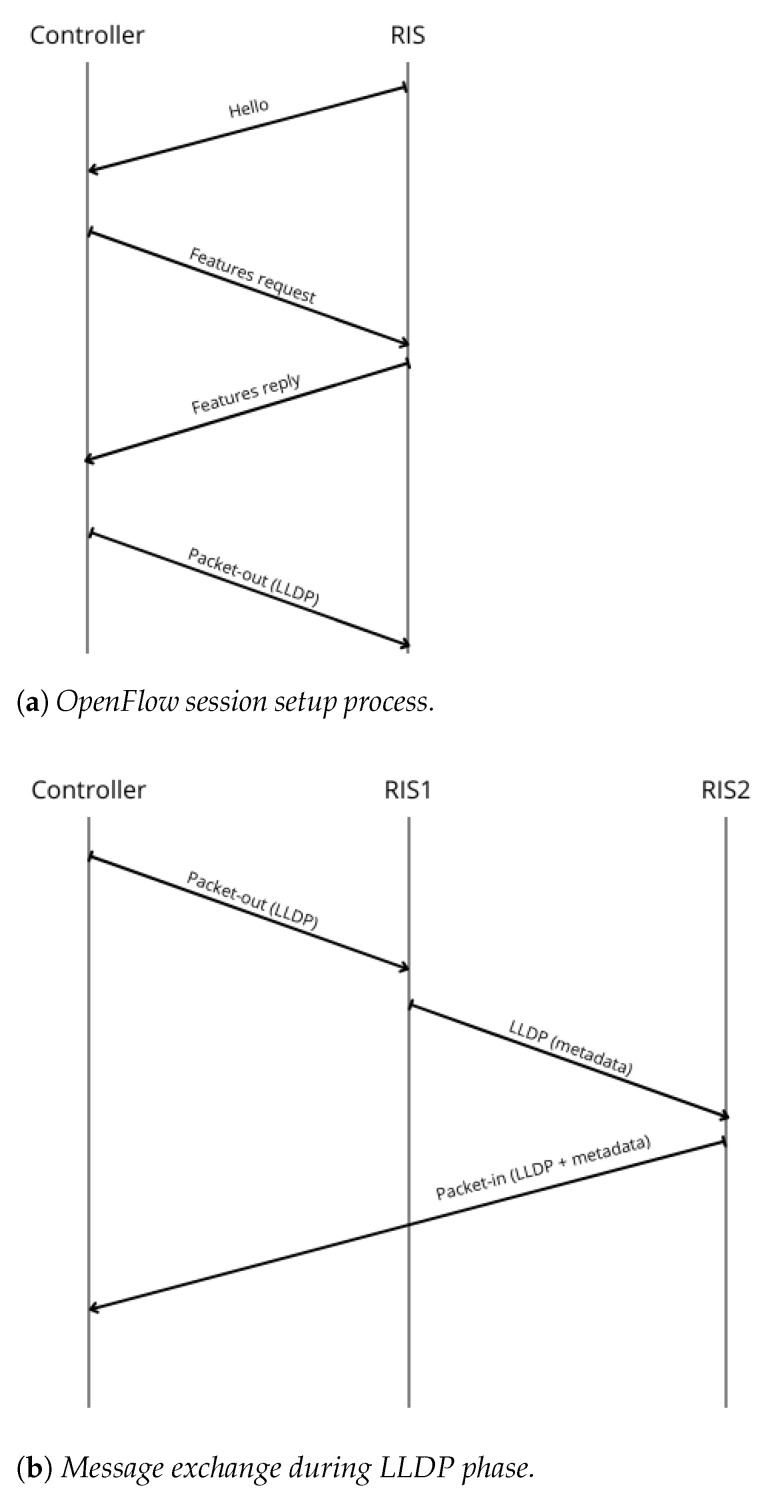
Proposed ND protocol.

The initial testing phase of the proposed system architecture focuses on the overall discovery phase, which is divided into two main steps: Switch discovery and Host discovery. The former involves the discovery of SDN Switches (i.e., RIS), which takes place prior to any message exchange, while the latter is related to IoT Devices (that are UEs in cellular terminology), and occurs as a result of the communication process. The objective of the ND phase is, therefore, to construct and maintain a global representation of the network topology, capturing the nodes and their interconnections in the form of a graph, such as the one illustrated in Figure 5. In creating the graph that represents the underlying network infrastructure, every time an edge that connects two RISs is detected, a weight can be assigned to that edge, usually the RSSI representing the signal strength along that edge.

**Figure 6:**
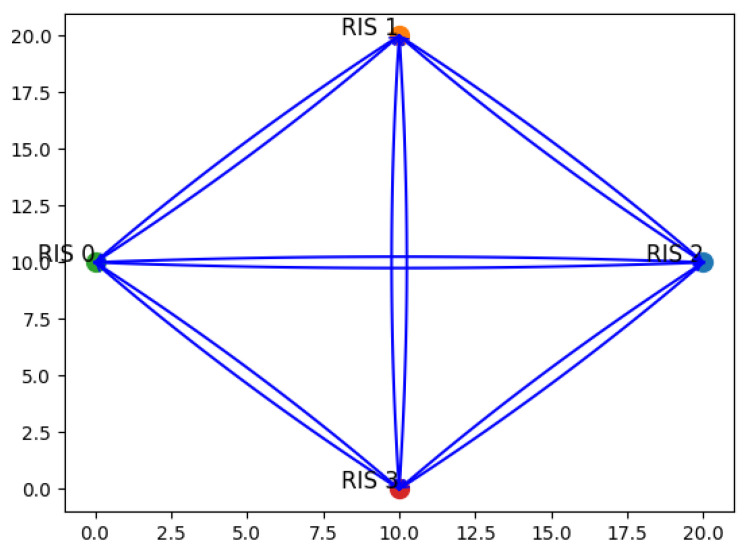
Network topology graph from switch discovery.

**Figure 7:**
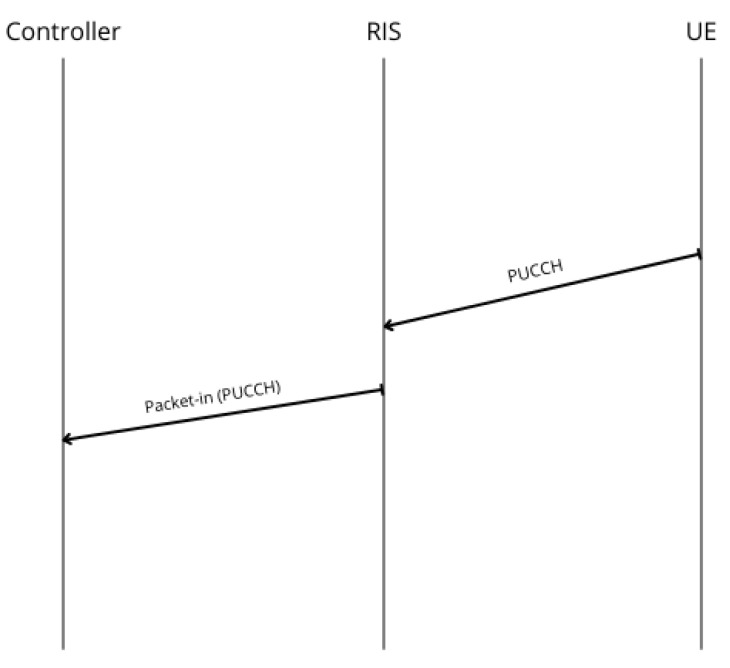
Message exchange during UE discovery phase.

**Figure 8:**
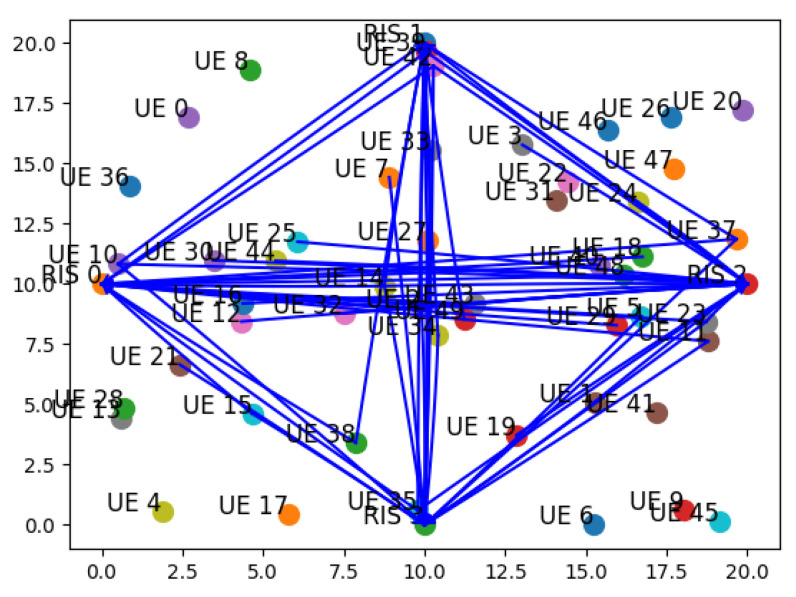
An example of a complete network topology graph also pointing out the presence of UEs.

1. **Switch discovery**: The communication process begins with the establishment of the OF session between the RIS and the SDN Controller (Figure 5a). As part of the standard OF handshake, the RIS (functioning as an OF-enabled Switch) initiates the session by sending a Hello message to the Controller. Upon receiving it, the Controller sends a Features Request message to query the RIS capabilities. The RIS responds with a Features Reply, providing essential information such as its ID and the list of available OF ports (mapped to RIS beams). This initial exchange of control messages constitutes the session setup phase, allowing the Controller to recognize and manage the RIS as part of the SDN topology.
  Once the session is established, the SDN Controller initiates the ND phase (Figure 5b). Leveraging the knowledge of each Switch (i.e., RIS) ports, it sends a Packet-Out message containing an LLDP packet, having previously set the RIS to broadcast it through all its active ports. Upon its reception, any Switch appends its own identifier and port information to the packet and retransmits it across its interfaces. When this message is in turn received by another RIS, it sends a Packet-In message to the Controller, including metadata about the source device. This enables the Controller to infer direct wireless links between devices and to construct an accurate view of the network topology. Figure 6 presents the resulting graph, where RIS components and all feasible links within the network are pointed out.
2. **Host discovery**: Host discovery is dynamically triggered during the simulation when UE initiates communication by transmitting a PUCCH packet. For each RIS deployed in the network, it is evaluated whether the signal was received based on the RIS position and beam configuration. Each RIS that receives the signal is able to generate a Packet-In message and forwards it to the SDN Controller via the OpenFlow channel. This message contains information about the UE and the associated RIS, effectively notifying the SDN Controller of the detected link. Upon receiving this information, the Controller updates the network topology to add the discovered association between the UE and the RIS. Figure 7 illustrates the described message exchange, while Figure 8 represents all the possible links between RISs and UEs.

#### 3.3.2. Coverage Analysis

The preliminary tests conducted in the simulation campaign focus on coverage analysis to evaluate the network’s reachability under different scenario configurations. It could be noticed that it represents a particular case of the optimization problem (1) where $c_{ij}=1,\forall i,j$.

It was assumed that UEs could either be in LoS or NLoS conditions with the gNodeB, where UEs in LoS can directly communicate with the gNodeB without requiring any additional support. Conversely, UEs in NLoS conditions cannot establish a viable direct link and are therefore able to communicate only if their path is properly assisted by a RIS. In this context, a user is defined as reachable if it is served by at least one RIS beam, meaning that the RIS effectively extends the coverage region of the gNodeB by enabling connectivity where the direct channel is blocked.

It is important to clarify that, in our analysis, we assumed that RISs only support UEs in NLoS conditions, while users with a direct LoS path do not require RIS. This allows us to isolate and quantify the RIS contribution in the most critical propagation scenarios, namely those ones where conventional communication would otherwise fail. However, this assumption does not exclude the possibility of employing RISs even when the UE is already in LoS with the gNodeB. In practice, RISs may still be beneficial in LoS scenarios, for example, to improve received signal strength, enhance beamforming gain, mitigate interference, or assist in mobility events. These use cases, while relevant, fall outside the scope of the present study and were therefore not considered in our performance evaluation.

Under the adopted assumptions, overall coverage is defined as follows: (i) in scenarios without RISs, coverage comprises solely the set of UEs that are in LoS with the gNodeB; and (ii) in scenarios where RISs are deployed, coverage includes both the UEs in LoS with the gNodeB and the UEs in NLoS that are brought into coverage through a LoS path with at least one RIS. This definition reflects the role of the RIS as an extension mechanism that compensates for the absence of a direct gNodeB–UE link.

**Figure 9:**
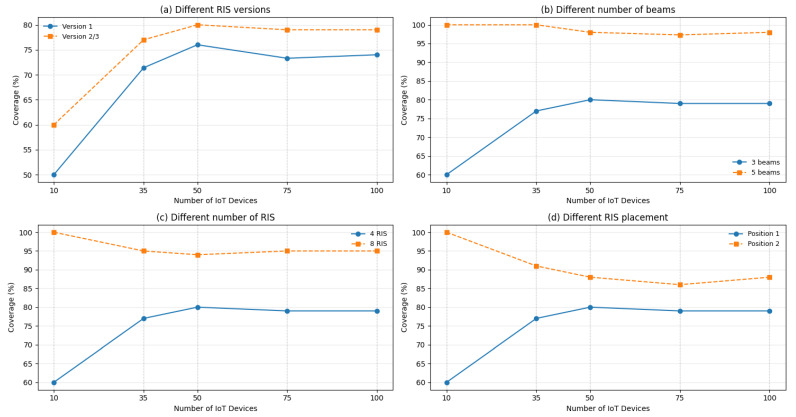
Network coverage comparison for different scenarios.

The first aspect taken into account when investigating network coverage is the version of RIS used. As mentioned in Section 2.2, version 1 of the RIS can reflect the received signal along a single, fixed beam direction, without any capability to alter or reconfigure the reflection path. In contrast, version 2 and 3 provide increasingly advanced control capabilities, allowing the reflected signal to be steered toward one beam selected from a discrete set (version 2) or even multiple beams simultaneously (version 3). This additional flexibility significantly enhances the effective coverage footprint. By being able to select and, in the case of version 3 RIS, to replicate the direction of reflection, these more advanced RISs can cover different areas or serve multiple users that would otherwise remain uncovered due to limitations of version 1 RIS. As shown in Figure 9a, while both curves follow a similar trend, the more complex RIS consistently outperforms the simpler one. For low user densities, the improvement reaches up to 10%, mainly due to the capability of version 2 and 3 to adapt their beams to the spatial distribution of users. As density increases, the gain becomes more moderate but remains stable around 4–5% points, still highlighting the benefit of increased configurability. These results confirm that adopting more advanced RIS versions leads to a larger and more flexible coverage region, enhancing the percentage of users that can be effectively served.

Finally, it is worth noting that, despite the potential advantages of both version 2 and 3, in this work we focus exclusively on version 2 RISs. This choice is motivated by the need to keep the simulation framework tractable, as implementing full multi-beam capabilities (version 3) would require a more complex control and resource allocation model that falls beyond the scope of the present study. Thus, from this point onward, the data presented always refer to RIS version 2.

The next step was to compare how the results change when the RIS operates with five beams, instead of three. Since each beam represents a distinct coverage direction, increasing the number of beams allows a single RIS to cover a larger area. Indeed, with only three beams, the coverage is more limited, as each RIS can only redirect the signal in a few predefined directions. On the other hand, with five beams, RIS can reflect the signal towards more UEs, statistically increasing the reachability of previously uncovered areas. In Figure 9b, the results obtained considering four version 2 RISs modifying the number of beams are pointed out. The curve representing the RIS with five beams displays a trend that is substantially different from that of the other curve, with a coverage percentage that remains between 97% and 100%. In contrast, the coverage achieved by the RIS with three beams exhibits much greater variability, spanning 60% to 80%.

Another aspect that has been taken into consideration is the number of RISs used. Figure 9c shows how coverage changes by modifying the number of RISs from four to eight. Here, the curve representing the coverage trend of the 8-RIS setup shows an average improvement of 20% with respect to the four-RIS case.

Finally, an important parameter is the positioning of the RIS within the scenario, since it is considered along the RPSP. According to a simple heuristic, the RISs have been placed in the center of each of the four walls. An alternative approach could be to position the four RISs in the corners of the room instead. Figure 9d shows a comparison on this aspect, where “Position 1” refers to the one in which the RISs are positioned in the center of the wall and “Position 2” refers to the one in which they are in correspondence with the edges. In this scenario, the more remarkable difference is observed for lower user densities, with coverage ranging from 60% in Position 1 to 100% in Position 2. As the number of users increases, the coverage performance stabilizes, with only a few percentage points of difference.

**Figure 10:**
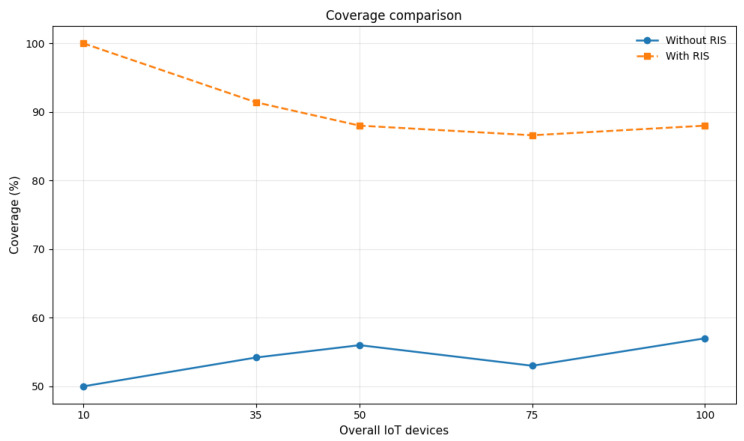
Network coverage comparison with and without RIS deployment for different values of IoT devices.

In general, we can compare the network coverage in the original case, without RISs, with a scenario where, for example, four version 2 RISs with three beams are positioned at the corners of the scenario. Figure 10 clearly illustrates the gain achieved through the presence of the RIS. In the absence of RIS, coverage remains relatively low and stable, ranging between 50% and 60% across the user range. In contrast, the deployment of RIS significantly improves coverage, with values between approximately 80% and 100%. The coverage advantage is particularly pronounced at low user densities, where the percentage reaches its maximum, and gradually decreases as the number of users increases.

The previously presented results demonstrate how network coverage improves as the RIS parameters are optimized. Specifically, optimizing the number of RISs, increasing the number of beams per RIS, and properly placing the RIS jointly contribute to enhancing the coverage. However, this improvement comes with an increased system complexity. Moreover, the positioning of RISs plays a crucial role, as different configurations can significantly impact overall performance. These findings highlight the trade-off between coverage enhancement and implementation complexity, emphasizing the need for a balanced design approach, and motivating for a proper optimization as it is pointed out in the following.

#### 3.3.3. RIS Placement and Selection Analysis

**Figure 11:**
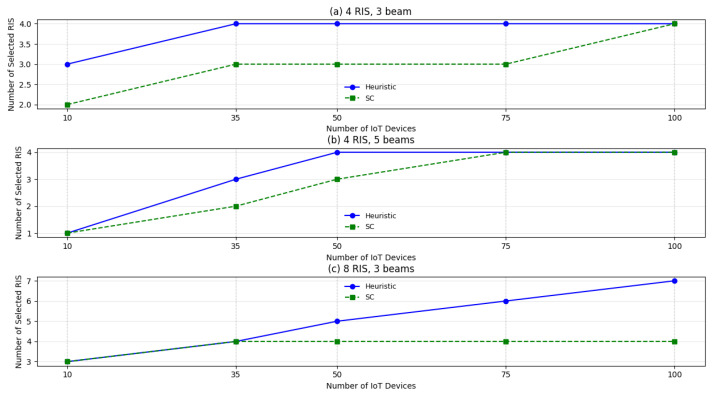
RIS selection comparison between heuristic approach and Set Covering approach in different scenarios.

As mentioned in Section 2.4, the RPSP represents an important aspect to be investigated. This problem can be interpreted in two complementary ways: *placement*, which concerns determining the optimal location for the RIS deployment; and *selection*, which involves identifying which pre-deployed RIS should be activated to facilitate communication. Therefore, various methodologies have been used and compared for the selection and placement of RIS within the considered scenario. Three approaches have been tested and compared and in Figure 11 it is possible to observe some comparative results analysis.

In the scenario with four RISs and three beams per RIS sketched in Figure 11a, the heuristic method tends to activate unnecessary RISs, where the maximum value is represented by four devices. The SC approach, instead, maintains a lower number of active RISs across different scenarios and approaches the heuristic solution only for very high IoT densities, thus ensuring a more resource-effective system configuration.

A similar behavior can be observed when considering four RISs with a higher beamforming capability, i.e., five beams per RIS (Figure 11b). In this case, both methods start with selecting a single RIS for low IoT density scenarios. However, as the number of IoT devices increases, the heuristic solution sharply saturates, involving all the four available RISs. The SC scheme, on the other hand, increases more gradually and only reaches the maximum RIS utilization in very dense networks, effectively trading-off performance and complexity.

Finally, in the scenario with eight RISs and three beams each, as reported in Figure 11c, the heuristic solution quickly increases the number of active RISs, activating seven RISs for serving 100 IoT devices. Conversely, the SC approach requires at most four RISs, regardless of further increases in IoT devices.

Another key feature of the SC approach is its ability to preserve redundancy, enabling users to be served by multiple selected RISs. This allows the system to select an optimized path based on specific criteria.

**Figure 12:**
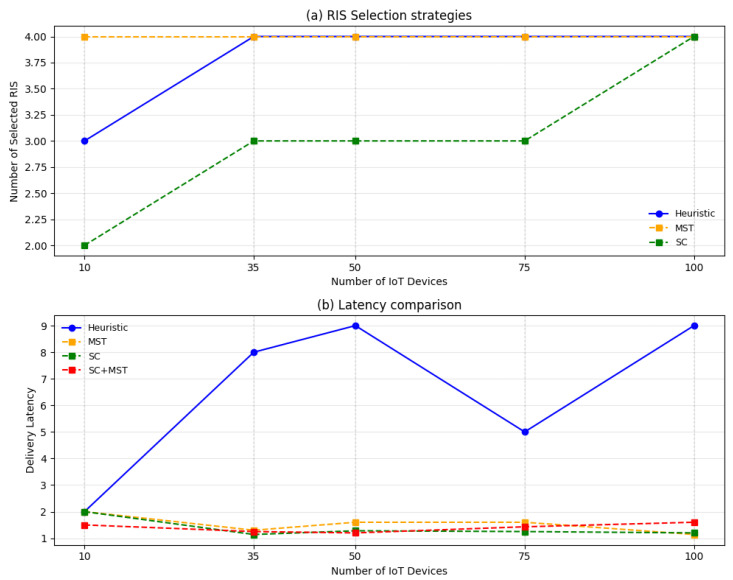
Comparison of RIS selection and latency performance for different optimization strategies.

Figure 12a illustrates the trend of MST approach when applied to the selection of RISs. As minimizing the number of selected RISs is not the primary objective of the MST formulation, it can be observed that its values are generally higher than those obtained with the SC-based strategy. For this reason, particular attention has been given to the comparison of delivery latency, as shown in Figure 12b. In fact, the MST algorithm does not aim to reduce the number of active RISs, but it identifies paths with the minimum overall cost, which, in this context, is related to the latency. As a consequence, while SC is effective in reducing the deployment overhead, MST focuses on improving transmission efficiency. Furthermore, their sequential combination (SC + MST) provides a balanced solution that jointly benefits from both perspectives: the SC approach provides the advantage of selecting the minimum number of RISs, while the MST strategy eliminates redundancy by establishing the shortest and most efficient paths for each user.

In conclusion, based on the tests and comparisons, we can infer that both algorithms (Algorithms 2 and 3) solve the RPSP, but the SC approach is a more suitable candidate for determining the minimum number of RISs required within the scenario, as well as for their positioning and configuration to enable communication among the network nodes, while the MST approach, integrated with a proper cost model, selects the subset of RISs that optimize the overall communication performance.

## 4. Conclusions and Future Work

In this paper, we present an SDN-based framework for the management of RIS devices in Industrial Mobile IoT 6G scenarios. The proposed architecture integrates RISs as OpenFlow-compatible network elements, enabling centralized programmability and seamless coordination through the SDN controller. Simulation results demonstrated that the joint adoption of SDN and RIS technologies significantly enhances network coverage and connectivity, especially under NLoS conditions, achieving a coverage that remains between 80% and 100% of the overall displaced IoT devices. Furthermore, the proposed optimization algorithms based on both SC and MST formulations proved effective in reducing the number of active RISs, while maintaining or improving service quality, thereby balancing network performance and deployment costs.

Despite these promising results, several challenges and open issues remain. First, our current evaluation focuses primarily on RIS version 2, while the more advanced version 3, capable of supporting multi-beam operations and offering higher configurability, has not yet been fully implemented or tested within our framework. Extending the architecture and optimization algorithms to accommodate advanced RIS or even more complex models represents a necessary direction for future works. Moreover, all the results were obtained through simulation; therefore, validating the proposed system using real RIS hardware and an actual SDN-enabled emaulated testbed would provide valuable insights into practical constraints, hardware limitations, and real-world performance.

From a networking perspective, the reliance on OpenFlow for RIS control, although useful for prototyping, introduces overhead and may not represent the most efficient solution for large-scale or latency-sensitive deployments. As a consequence, replacing OpenFlow with lighter, more specialized southbound protocols, as NETCONF or P4, could significantly improve scalability and responsiveness, as it could be assessed by emulating the overall system.

## Figures and Tables

**Table 1:** Main simulation parameters.

Parameter	Value/Range
Scenario size	20×20×4 [m^3^]
Number of gNodeB	1
Number of UEs	10–100
Number of RIS	4–8
Obstacles	16

## Data Availability

Data is contained within the article.
